# Patient Weight and Chemoprophylaxis in Abdominoplasty: Does It Result in More Bleeding Events?

**DOI:** 10.1007/s00266-024-04220-w

**Published:** 2024-07-09

**Authors:** Ahmed Ibrahim, Ingrid Steinvall, Sherif Elawa, Mohamed A. Ellabban, Mostafa K. Mohamed, Moustafa Elmasry, Islam Abdelrahman

**Affiliations:** 1 Nottingham University Hospitals, Nottingham, England; 2 Department of Hand Surgery, Plastic Surgery and Burns and Department of Biomedical and Clinical Sciences, Linköping University, 58185 Linköping, Sweden; 3 Plastic and Reconstructive Surgery Unit, Department of Surgery, Faculty of Medicine, Suez Canal University, Ismailia, Egypt

**Keywords:** Abdominoplasty, Chemoprophylaxis, Post-massive weight loss, Haematoma, Bleeding, Venous thromboembolism

## Abstract

**Background:**

Bariatric surgery has gained popularity in recent decades as an effective treatment for obesity. Abdominoplasty is one of the most often performed aesthetic procedures all over the world. In post-bariatric patients undergoing abdominoplasty, the diameter size and number of the abdominal wall perforators increase proportionally with increased body weight. Postoperative complications that may occur are haematoma, and venous thromboembolism (VTE). In plastic surgery procedures VTE prophylaxis grades vary due to the lack of consensus and clear guidelines. The aim of this study was to explore the frequency of postoperative bleeding and VTE in patients undergoing abdominoplasty and to explore the risk factors associated with major bleeding.

**Methods:**

A retrospective single-centre study of adult patients who were operated on by abdominoplasty between 2011 and 2020. Chemoprophylaxis including low molecular weight heparin (LMHW) was recommended when the operating time exceeded 2 h.

**Results:**

A total of 102 patients were included. There were no patients with VTE. Eight patients were re-operated for major haematoma. The weight loss (peak weight to weight before the abdominoplasty) was 14.4 kg larger in the re-operation group (*p *= 0.03). Eighty-eight percent in the re-operation group and 67% in the other group were treated with LMWH (*p *= 0.43). Multivariable logistic regression showed that with each decrease from the peak in BMI kg/m^2^ the risk of re-operation for major haematoma was increased by 22% (*p *= 0.02).

**Conclusion:**

Abdominoplasty in patients after massive weight loss has a higher risk of postoperative bleeding. Having a clear protocol for chemoprophylaxis should be considered.

**Level of Evidence III:**

This journal requires that authors assign a level of evidence to each article. For a full description of these Evidence-Based Medicine ratings, please refer to the Table of Contents or the online Instructions to Authors www.springer.com/00266.

## Introduction

Bariatric surgery has gained popularity in recent decades as an effective treatment for obesity and its related comorbidities. This has resulted in an increasing number of patients seeking body contouring procedures due to the unwanted excess skin. Abdominoplasty is considered one of the most performed aesthetic procedures all over the world. The operation entails the correction of skin laxity after pregnancy or weight loss. The aim of the procedure is to restore a flat contour of the abdomen in addition to a natural, appealing umbilicus, and finally a well-hidden abdominal scar. A challenge in post-bariatric patients undergoing abdominoplasty is that the diameter size and number of the abdominal wall perforators increase proportionally with increased body weight. This change remains obvious even if the patient returns to normal weight after bariatric surgery [1, 2]. Moreover, these blood vessels seem to be more fragile than those of controls [3].

Postoperative complications that may occur after this kind of operation are haematoma, seroma, wound infection, dehiscence, contour irregularities, and venous thromboembolism (VTE) [4].

VTE is considered one of the most serious and possible life-threatening complications for surgical patients. Reports of VTE vary in plastic surgery between 0.49% in patients who have undergone facelifts and 9% in those who have had belt lipectomy [4, 5]. VTE prophylaxis grades in plastic surgery procedures vary amongst different institutes due to the lack of consensus and clear guidelines. To our knowledge, there is only consensus regarding the use of mechanical prophylaxis [6]. However, there is an ongoing debate about using pharmacological prophylaxis from a risk/benefit perspective [5]. Combined mechanical prophylaxis and chemoprophylaxis in the form of low molecular weight heparin have shown good results, particularly in higher-risk patients [5]. On the other hand, some studies have shown an increased risk of postoperative bleeding [7, 8].

The frequency of postoperative bleeding after abdominoplasty varies between studies [4], but this has not been studied in a European cohort. Our hypothesis is that chemoprophylaxis would increase the risk of postoperative bleeding and that the excessive weight loss prior to the procedure would increase the risk of bleeding due to larger abdominal wall perforators.

The aim of this study was to explore the frequency of postoperative bleeding and VTE in patients undergoing abdominoplasty and to explore the risk factors associated with major bleeding.

## Methods

This was a retrospective single-centre study done at the Plastic Surgery Department at Linköping University Hospital, Linköping Sweden. Inclusion criteria: adult patients who had had abdominoplasty between 2011 and 2020. Exclusion criteria: those who had had abdominoplasty for reasons other than a sagging abdomen, for example, scar correction or local skin disease in the lower abdomen.

In our department, abdominoplasty is offered to patients who have skin laxity and folding in the lower abdomen with a minimal distance of 3 cm. The patients should have a maximum BMI of 25. Classic abdominoplasty is done in most cases while the fleur-de-lis method is sometimes done if the patients have skin laxity in the upper and lateral parts of the abdomen. All surgeons follow the same routine regarding the operation design and surgical residents are supervised by a consultant.

Most of the patients in the study were admitted to the hospital ward for one night after the operation. We usually follow a local guideline regarding VTE, including pneumatic compression devices and graduated compression stockings, for all patients. Chemoprophylaxis including LMHW was recommended when the operating time exceeded 2 h. Chemoprophylaxis was administered 6 h after the end of the operation for 7 days. An early mobilisation routine was strictly followed after the operation. Drainage tubes were removed when the amount of drainage was less than 50 ml/24 h. A follow-up visit to a specialised nurse was scheduled within 10 days after the operation. Another visit was scheduled to the operating surgeon within 6 months to evaluate the final outcome.

As all the operations done in our hospital are covered by the national insurance system, it is not permitted to combine liposuction with abdominoplasty according to the Swedish national guideline for official health care.

Haematoma exploration was usually done on clinical suspicion such as unstable vital parameters, tense and tender abdomen, and rapid decrease in haemoglobin concentration. Caprini score was not used as a guide for VTE prophylaxis. The one presented in the study was retrospectively calculated by the authors.

Variables: age, body mass index (BMI) and peak body weight before weight loss, body mass index (BMI), and body weight decrease between the peak and the abdominoplasty operation, comorbidities, sex, operation time, amount of intraoperative bleeding, weight of recessed tissue, major haematoma (haematoma that required surgical exploration), minor haematoma, wound infection, seroma, postoperative treatment with low molecular weight heparin (LMWH), previous bariatric surgery, DVT risk by Caprini score [9], previous smoking, use of oral contraceptive, ASA class, and blood type.

Two subgroup analyses were done by grouping the patients according to previous bariatric surgery; and haematoma exploration.

This study was approved by the Swedish Ethical Review Authority (2022-02051-01).

### Statistics

Data were analysed with the help of STATISTICA 13 (TIBCO, software Inc.). Data are presented as median (IQR), mean (SD) and *n* (%) unless otherwise stated. Mann Whitney U, *t*-test, chi-squared and Fisher exact tests were used, as well as logistic multivariable regression. Statistical significance was set at *p *< 0.05.

## Results

A total of 102 patients were included (Tables 1 and 2). There were no patients with VTE after the abdominoplasty. Eight patients were re-operated for major haematoma (haematoma exploration) (Tables 1, 2). The weight loss was 14.4 kg larger in the re-operation group (*p *= 0.03) and the BMI decrease was 4.1 kg/m^2^ larger (*p *= 0.07) (Table 1). Seven of the eight (88%) in the re-operation group and 63/94 (67%) in the other group were treated with LMWH (*p *= 0.43) (Table 2). Tables 3 and 4 show the same variables grouped by whether they had bariatric surgery or not.

**Table 1:** Details of the patients grouped by postoperative bleeding

	All	No re-operation	Re-operation group	*p* value	
Patients	102	94	8		
Age, years	45.5 (12.1)	45.7 (12.1)	42.5 (12.2)	0.48	*t* test
Sex, female	93 (91)	87 (93)	6 (75)	0.15	Fisher
Height, m	1.65 (1.62–1.71)	1.65 (1.62–1.71)	1.69 (1.63–1.77)	0.23	MWU
BMI at abdominoplasty	25.3 (24.3–27.0)	25.3 (24.3–26.9)	26.1 (23.2–27.2)	0.82	MWU
BMI peak	41.5 (38.2–44.8)	41.2 (38.2–44.3)	44.9 (40.6–50.3)	0.12	MWU
BMI decrease	16.5 (6.1)	16.1 (5.9)	20.2 (7.8)	0.07	*t* test
Weight pre-op, kg	69.0 (64.0–76.0)	68.0 (64.0–76.0)	72.3 (64.7–86.0)	0.56	MWU
Weight peak, kg	114 (103–127)	114 (102–126)	120 (112–163)	0.12	MWU
Weight loss, kg	45.8 (17.8)	44.6 (16.8)	58.9 (23.5)	0.03	*t* test
Bariatric surgery	81 (79)	74 (79)	7 (88)	1.00	Fisher
Years after bariatric surgery^a^	3.0 (3.0–5.0)	3.0 (3.0–5.0)	3.0 (3.0–4.0)	0.91	MWU
Comorbidity					
Hypertensive	18 (18)	16 (17)	2 (25)	0.63	Fisher
Myocardial infarction	1 (1)	1 (1)	0	1.00	Fisher
Diabetes	10 (10)	9 (10)	1 (13)	0.58	Fisher
Renal disease	2 (2)	2 (2)	0	1.00	Fisher
Cancer	6 (6)	6 (6)	0	1.00	Fisher
VTE/coagulation disorder	5 (5)	5 (5)	0	1.00	Fisher
Systemic diagnosis	4 (4)	4 (4)	0	1.00	Fisher
Pulmonary disease	5 (5)	5 (5)	0	1.00	Fisher
Smoker	15 (15)	13 (14)	2 (25)	0.33	Fisher
Oral contraceptive use	3 (3)	3 (3)	0 (0)	1.00	Fisher

Data are presented as mean (SD), median (25th–75th centile), or *n* (%). *T* test, Mann–Whitney U test, and Fisher’s exact test

^a^Calculated on patients with a previous bariatric surgery

**Table 2:** Abdominoplasty-related variables and risk-related variables, grouped by postoperative bleeding

	All	No re-operation	Re-operation group	*p* value	
Patients	102	94	8		
Operation time, minutes	120 (95–140)	120 (98–145)	114 (90–128)	0.41	MWU
Excision, gram	1350 (800–1800)	1370 (800–1705)	1315 (695–2000)	0.77	MWU
Bleeding (abdominoplasty), ml	50 (0–150)	50 (0–150)	50 (50–212)	0.50	MWU
Haematoma (no exploration)	9 (9)	9 (10)	0 (0)	1.00	Fisher
Seroma	9 (9)	9 (10)	0 (0)	1.00	Fisher
Infection (inclusive cellulitis)	8 (8)	8 (9)	0 (0)	1.00	Fisher
LMWH	70 (69)	63 (67)	7 (88)	0.43	Fisher
Risk group by Caprini score				0.55	Chi Squared
High risk	16 (16)	15 (16)	1 (13)		
Moderate risk	73 (72)	68 (72)	5 (63)		
Low risk	13 (13)	11 (12)	2 (25)		
ASA class				0.36	Chi Squared
ASA I	41 (40)	36 (38)	5 (63)		
ASA II	54 (53)	51 (54)	3 (38)		
ASA III	7 (7)	7 (7)	0 (0)		
Blood type				0.10	Chi Squared
B	14 (14)	13 (14)	1 (13)		
A	48 (47)	42 (45)	6 (75)		
O	36 (35)	36 (38)	0 (0)		
AB	4 (4)	3 (3)	1 (13)		

Data are presented as mean (SD), median (25th–75th centile), or *n* (%). *T* test, Mann–Whitney U test, Chi Squared, and Fisher’s exact test

**Table 3:** Details of the patients grouped by bariatric surgery

	Bariatric surgery	No bariatric surgery	*p* value	
Patients	81	21		
Age, years	44.9 (10.9)	47.6 (15.9)	0.36	*t* test
Sex, female	74 (91)	19 (90)	1.00	Fisher
Height, m	1.65 (1.62–1.70)	1.64 (1.62–1.75)	0.69	MWU
BMI at abdominoplasty	25.3 (24.3–26.9)	25.8 (24.3–27.3)	0.63	MWU
BMI peak	42.3 (39.5–46.9)	36.9 (28.3–40.3)	< 0.001	MWU
BMI decrease	18.0 (4.9)	8.9 (6.1)	< 0.001	*t* test
Weight pre-op, kg	69 (64–76)	68 (64–76)	0.78	MWU
Weight peak, kg	117 (106–128)	97 (73–114)	0.002	MWU
Weight loss, kg	50.0 (14.5)	25.1 (18.1)	< 0.001	*t* test
Years after bariatric surgery	3.0 (3.0–5.0)			
Comorbidity				
Hypertensive	16 (20)	2 (10)	0.35	Fisher
Myocardial infarction	1 (1)	0 (0)	1.00	Fisher
Diabetes	10 (12)	0 (0)	0.12	Fisher
Renal disease	1 (1)	1 (5)	0.37	Fisher
Cancer	3 (4)	3 (14)	0.10	Fisher
VTE/coagulation disorder	5 (6)	0 (0)	0.58	Fisher
Systemic diagnosis	2 (2)	2 (10)	0.19	Fisher
Pulmonary disease	5 (6)	0 (0)	0.58	Fisher
Smoker	13 (16)	2 (10)	0.73	Fisher
Oral contraceptive use	2 (2)	1 (5)	0.50	Fisher

Data are presented as mean (SD), median (25th–75th centile), or *n* (%). *T* test, Mann–Whitney U test, and Fisher’s exact test

**Table 4:** Abdominoplasty-related variables and risk-related variables, grouped by bariatric surgery

	Bariatric surgery	No bariatric surgery	*p* value	
Patients	81	21		
Operation time, minutes	125 (100–145)	100 (80–115)	0.006	MWU
Excision, gram	1400 (888–1800)	800 (500–1500)	0.06	MWU
Bleeding (abdominoplasty), ml	50 (0–150)	50 (0–73)	0.17	MWU
Major haematoma (exploration)	7 (9)	1 (5)	1.00	Fisher
Haematoma (no exploration)	7 (9)	2 (10)	1.00	Fisher
Seroma	6 (7)	3 (14)	0.39	Fisher
Infection (inclusive cellulitis)	8 (10)	0 (0)	0.39	Fisher
LMWH Y	60 (74)	10 (48)	0.02	Chi Squared
Risk group by Caprini score			0.19	Chi Squared
High risk	10 (12)	6 (29)		
Moderate risk	60 (74)	13 (62)		
Low risk	11 (14)	2 (10)		
ASA class			0.21	Chi Squared
ASA I	29 (36)	12 (57)		
ASA II	46 (57)	8 (38)		
ASA III	6 (7)	1 (5)		
Blood type			0.37	Chi Squared
B	9 (11)	5 (24)		
A	39 (48)	9 (43)		
O	29 (36)	7 (33)		
AB	4 (5)	0 (0)		

Data are presented as mean (SD), median (25th–75th centile), or *n* (%). *T* test, Mann–Whitney U test, Chi Squared, and Fisher’s exact test

**Fig. 1:**
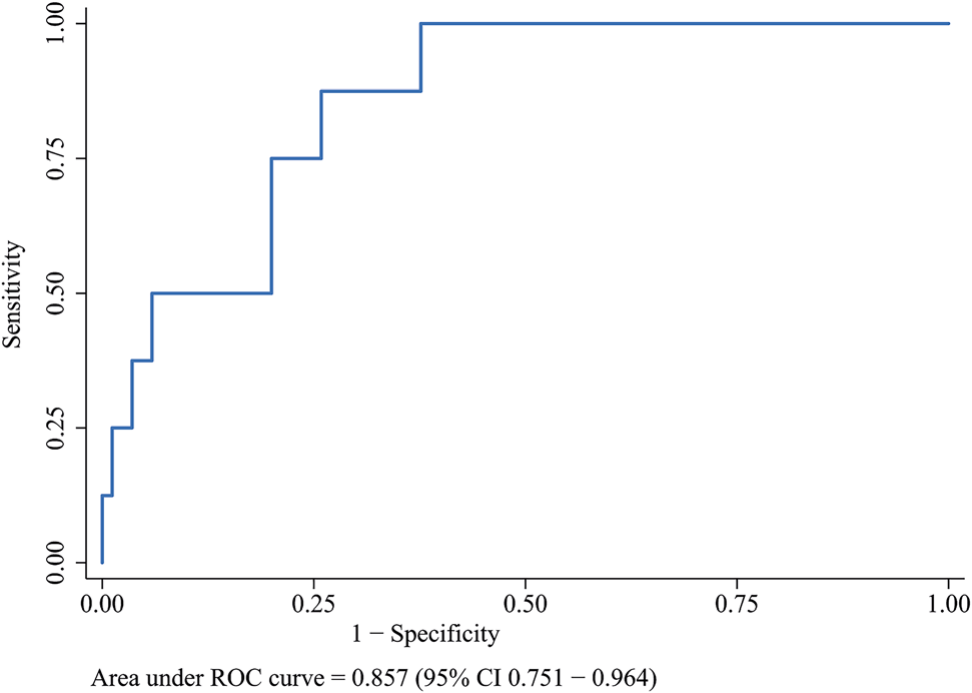
The AUC ROC curve of the logistic regression model for postoperative bleeding (major haematoma with re-operation)

The multivariable logistic regression showed that with each decrease in BMI kg/m^2^, the risk of re-operation for major haematoma was increased by 22% (*p *= 0.02), independent of treatment with LMWH and the other risk factors. Treatment with LMWH increased the risk 3.4 times although that result was not significant (*p *= 0.06) (Table 5 and Figure 1). Testing only those two variables together showed that there was still an independent effect of BMI decrease (coefficient 0.14, *p *= 0.048), while the effect of LMWH was not significant (coefficient 1.49, *p *= 0.20).

**Table 5:** Logistic regression for post-operative bleeding (major haematoma with re-operation)

	Coefficient	*p* value	OR	95% CI
BMI decrease	0.22	0.02	1.24	1.04–1.49
LMWH	3.39	0.06	29.78	0.90–986.10
Age, years	0.04	0.52	1.04	0.92–1.18
Bariatric surgery	− 0.88	0.56	0.42	0.02–8.07
Caprini group^a^			1.00	
Moderate risk	− 1.39	0.35	0.25	0.01–4.69
High risk	− 0.46	0.83	0.63	0.01–46.73
Operation time, minutes	− 0.02	0.18	0.98	0.95–1.01
ASA class II-III (I is reference)	− 2.13	0.06	0.12	0.01–1.07
Constant	− 5.45	0.12		

Multivariable model *p *= 0.09, Pseudo *R*^2^ 0.25, *n *= 93, ROC AUC 0.857 (95% CI 0.751–0.964)

^a^Low risk is reference

Table 6 shows an overview, of the number of patients who were re-operated for bleeding complications grouped by Caprini score and LMWH treatment.

**Table 6:** Number of patients who were re-operated for bleeding complications grouped by Caprini score and LMWH treatment

	Bleeding		No bleeding	
Caprini score	LMWH	No LMWH	LMWH	No LMWH
Low risk	1	1	4	7
Moderate risk	5	0	49	19
High risk	1	0	10	5

## Discussion

### Abdominoplasty and Chemoprophylaxis

The uncertainty remains for each abdominoplasty case in deciding when to start LMWH and trying to balance between avoiding VTE and postoperative bleeding. There is no evidence-based protocol for VTE prophylaxis in plastic surgery patients [10].

The consequences of VTE and postoperative bleeding can be life-threatening. However, their magnitude is different, given that bleeding can be addressed and managed by transfusion or return to theatre for control. On the other hand, VTE can go unnoticed initially and can be life-threatening in 10% of patients with pulmonary embolism with a more severe long-term impact that is very difficult to reverse [11, 12].

A systematic review of 1596 patients showed that chemoprophylaxis was neither associated with a reduced risk of VTE in abdominoplasty procedures 0.89% [6/671] versus 0.34% [3/885] nor with an increased risk of postoperative bleeding. It also highlighted the importance of stratifying patients according to risk to guide decision-making [4].

In this cohort, overall postoperative bleeding was 8% [8/102] compared with 1.9% (25/1596) in the systematic review. On the other hand, no cases developed VTE compared with the incidence of VTE reported, which was 0.56% (9/1596) in the form of DVT and PE [4].

Hatef et al. reported a 5% risk of VTE in abdominoplasty and 7.7% in circumferential abdominoplasty patients and had decreased incidence of DVT in circumferential abdominoplasty with using enoxaparin. However, they had a higher rate of bleeding that required transfusion. This may have been due to the timing of enoxaparin administration as they have been administering the enoxaparin 1–2 h preoperatively or intraoperatively or no later than 2 h postoperatively [13]. This is different from our practise of administering LMWH 6–8 h postoperatively.

### Abdominoplasty After Bariatric Surgery

A study by Shayan et al. looked at 67 patients by measuring the abdominal perforators by CT or intraoperatively and correlated the size of the perforators to the body weight. It concluded that the higher the BMI the bigger and more perforators and that those perforators remain the same size even after weight loss. It was quantified to be higher in number of perforators more than 1.5 mm by 2.6–3.3 folds and 1.2-fold larger in diameter of the five largest perforators [1]. Similar findings were described by Baptista et al. [6] who showed a higher number and bigger vessels in the adipose tissues of ex-obese patients in comparison with the control group.

This explains our findings that showed a significant difference and higher rate of postoperative bleeding that needed to return to theatre in the group that had higher weight loss (Table 1), despite no significant difference when we looked at the postoperative anticoagulant given.

This raised a question about the correlation between abdominoplasty in patients after massive weight loss such as after-bariatric surgery given the higher number and larger size of perforators in those patients. Should those patients be treated any differently from any other abdominoplasty in terms of VTE prophylaxis or intraoperatively?

Most plastic surgeons start chemical VTE prophylaxis six hours postoperatively, yet there is no evidence that this is the best for the patients. Similarly, whether preoperative heparin administration has any role [12, 14].

The common practise of administering LMWH 6–8 h postoperatively has proven to be safe and does not increase the risk of postoperative bleeding. Except for certain procedures that include massive dissection such as post bariatric body contouring, breast, and microsurgical procedures that showed a significantly higher rate of postoperative bleeding [15]. This comes in line with what we have noticed in our cohort for an increased rate of return to theatre due to bleeding in patients who had massive weight loss.

### Recommendations

Our findings showed an increased risk of postoperative bleeding in patients undergoing abdominoplasty post-bariatric surgery which can be attributed to the increased size and number of abdominal perforators in this group of patients. The fact that they were obese and underwent bariatric surgery put them at a higher risk group due to the associated risk of other comorbidities such as diabetes mellitus, hypertension, and ischemic heart disease with obesity generally. We recommend treating those patients with more care to tackle that increased risk of post-operative bleeding. This can be done by optimising the preoperative status such as haemoglobin level and treating any anemia even if it was mild or controlling other comorbidities before committing to that elective procedure. Intra-operatively, we recommend considering added careful measures in dealing with abdominal wall perforators by meticulous haemostasis by ligating those perforators, especially sizeable ones, and making sure the blood pressure at the time of haemostasis is equivalent to the patient’s normal blood pressure to avoid any rebound post-operative bleeding from those bigger perforators. Postoperatively, we recommend more frequent monitoring of those patients by the ward team, especially their vital status, and observing the drain output more frequently during their hospital stay, especially during the first 48 h. Future studies should investigate those and other different measures and their effect in tackling that increased postoperative bleeding risk in that group.

The results can provide a better basis for which surgical methods can be recommended for future patients. The results will also provide better knowledge about the relationships between different factors and the occurrence of complications, which can hopefully be used as a basis for preventing some future complications.

### Limitations

Out of the 102 patients, only eight patients had re-operation, which is a small cohort to use to correlate our findings. Because of the low incidence of VTE and postoperative bleeding after abdominoplasty, the sample size would need to be thousands of patients to gain sufficient power to establish definite evidence. This limitation is met in most of the articles addressing the same topic.

## Conclusion

In this cohort, performing abdominoplasty in post-massive weight loss patients has a higher risk of postoperative bleeding. Having a clear protocol of cautious chemoprophylaxis and more meticulous intraoperative haemostatic measures should be considered.
